# **β**-hydroxybutyrate enhances brain metabolism in normoglycemia and hyperglycemia, providing cerebroprotection in a mouse stroke model

**DOI:** 10.1177/0271678X251334222

**Published:** 2025-04-12

**Authors:** Deborah M Holstein, Afaf Saliba, Damian Lozano, Jiwan Kim, Kumar Sharma, James D Lechleiter

**Affiliations:** 1 Cell Systems and Anatomy, University of Texas Health San Antonio, San Antonio, Texas, USA; 2 Center for Precision Medicine, University of Texas Health San Antonio, San Antonio, Texas, USA; 3 Division of Nephrology, University of Texas Health San Antonio, San Antonio, Texas, USA

**Keywords:** Acute hyperglycemia, β-hydroxybutyrate, brain metabolism, *in vivo* brain imaging, oxygen consumption rate

## Abstract

Hyperglycemia in poorly controlled diabetes is widely recognized as detrimental to organ dysfunction. However, the acute effects of hyperglycemia on brain metabolism and function are not fully understood. The potential protective benefit of ketone bodies on mitochondrial function in the brain has also not been well characterized. Here, we evaluated the acute effects of hyperglycemia and β-hydroxybutyrate (BHB) on brain metabolism by employing a novel approach leveraging adenosine triphosphate (ATP)-dependence of bioluminescence originating from luciferin-luciferase activity. Oxygen consumption rate was measured in *ex vivo* live brain punches to further evaluate mitochondrial function. Our data demonstrate that brain metabolism in mice is affected by acute exposure to high glucose. This short-term effect of glucose exposure was reduced by co-administration with the ketone body BHB. Additionally, we investigated the functional relevance of BHB using an *in vivo* photothrombotic stroke model to assess its cerebroprotective effects in presence or absence of acute hyperglycemia. BHB significantly reduced infarct size in the brain stroke model, providing functional evidence for its protective role in the brain. These findings suggest that BHB may effectively mitigate the adverse effects of metabolic stress and ischemic events on brain metabolism and function.

## Introduction

The ketogenic diet has been established as a powerful treatment for epilepsy,^1^ and obesity ^2^ and cardiovascular diseases.^3^ Ketones are generated during starvation or states of reduced insulin action and may have beneficial attributes for cells. Ketones have been considered a “super-fuel” for the heart in a diabetic setting,^4,5^ but it remains unclear what the specific role of low carbohydrate/ketogenic diets is in other energetically demanding organs, such as the brain. Emerging evidence suggests that the macronutrient composition of diets, including low-carbohydrate or ketogenic diets, may influence brain function.^6,7^ For instance, a clinical trial demonstrated that long-term adherence to high-carbohydrate diets increased regional cerebral blood flow in brain regions associated with hunger and reward, such as the nucleus accumbens and hypothalamus, potentially contributing to weight regain.^8^ In a recent study, Wei et al. (2024) showed that a long-term ketogenic diet increased pro-inflammatory biomarkers in patients and increased brain senescence in mice’s brains, among other organs.^9^ These studies underscore the importance of understanding how macronutrient composition impacts brain energy balance and function, particularly under conditions of metabolic stress.

Glucose is an obligate fuel for neuronal cells, however glucose may be toxic at concentrations above 10 mM.^10^ Several studies demonstrated that high glucose levels are neurotoxic.^11,12^ Ketone bodies are also toxic at elevated levels.^13^ This toxicity at high concentrations of either carbohydrates or ketones underscores the importance of understanding how these affect brain metabolic balance. A key gap in knowledge concerns how acute changes in fuel availability, such as hyperglycemia and ketosis, impact brain metabolism. While studies have focused on isolated effects of glucose or ketones, few have examined their interaction *in vivo*. Mitochondrial dysfunction is linked to various neurological disorders^14^ and nutrients are considered beneficial if they support mitochondrial function.^15^ Thus, understanding the combined effects of glucose and ketones on mitochondrial activity in the brain is critical for optimizing dietary interventions such as the ketogenic diet.

In this study, we hypothesized that the ketone body BHB can fine-tune mitochondrial function in the brain, acutely mitigating effects associated with stressful metabolic conditions such as acute hyperglycemia or acute brain injury. To test this hypothesis, we developed a novel approach that combines *in vivo* live imaging with an established transgenic reporter mouse model.^16^ This model incorporates an astrocyte-specific promoter for glial fibrillary acidic protein (GFAP) along with an ATP-dependent luciferase enzyme, enabling us to dynamically assess the acute effects of glucose and BHB on brain mitochondrial activity. Luciferase assays have traditionally been used to estimate ATP levels *in vitro.*^17^ However, the novelty of our approach lies in the ability to monitor ATP levels and mitochondrial activity *in vivo,* using bioluminescence as a real-time measure of mitochondrial function. In these assays, luciferase activity depends on ATP hydrolysis, which acts on luciferin to produce bioluminescence. Fluctuations in ATP levels directly translate to changes in bioluminescence, providing an indirect measure of mitochondrial function in response to glucose and BHB. We also confirmed our data using *ex-vivo* tissue biopsies with oxygen consumption measurements. Moreover, to provide functional evidence linking metabolic modulation by BHB to brain health and further emphasize the potential relevance of ketone balance for cerebral resilience, we tested the BHB effect in brain protection from photothrombotic stroke. This model enables precise induction of localized ischemic injury and is widely used to study neuroprotection, making it appropriate for evaluating the acute effects of BHB on stroke-related metabolic stress.^18,19^ Our data indicate that the acute effects of metabolic stress or stroke on the brain can be mitigated by BHB.

## Methods

### Animals

All experiments including animal studies, were conducted in accordance with the Guide for the Care and Use of Laboratory Animals and approved by the Institutional Animal Care and Use Committee (IACUC) at the University of Texas Health Science Center at San Antonio. The study complies with the ARRIVE guidelines 2.0 for reporting animal experiments.

### In vivo bioluminescence imaging (BLI)

GFAP+GAPDH+ Dual-Glo mice (Jackson lab stock #009638) were fasted 1 day prior to BLI experiments. 3-5 months old, (n = 12 per group including 6 females and 6 males) were injected with 15 mg/kg Cycluc1,^20^ a D-luciferin analog and luciferase substrate (AOBIOUS cat# AOB1117) and anesthetized using 2.5% isoflurane inhalation. After ensuring appropriate anesthesia, mice were imaged using a Xenogen IVIS spectrum live-imaging system, a non-invasive *in vivo* imaging platform that detects and quantifies bioluminescence in real-time. The mice were injected subcutaneously with CycLuc1 in the scruff of the neck, and bioluminescence images were taken every minute for 35 minutes, without any delay.

### CycLuc1 and imaging

The dosage of Cycluc1, was chosen at 15 mg/kg, as this concentration was necessary to achieve a minimum detection threshold of 600 counts, ensuring reliable data quantification. At lower CycLuc1 concentrations, bioluminescence signals were too weak for accurate analysis. All mice were imaged using both an open filter and a 620 nm filter with an exposure time of 60 s. The field of view was set to FOV:C with a binning of 8. Signal quantification was performed in specific regions of interest (ROIs) by using fixed-size square ROIs throughout the experiments to maintain consistency across subjects and time points. Bioluminescent intensities were quantified as total photon flux (photons/second) in ROIs drawn manually over the brain area or over the feet area, with the latter serving as background control signals.

### Treatment groups

After 4 minutes of Cycluc1 imaging, mice were given a subcutaneous injection in the scruff of the neck using a 31-gauge BD syringe (BD cat# 328468) within a 10-second timeframe. The injections consisted of either 2.52 g/kg BHB (Sigma cat# H6501), 2 g/kg glucose (a high glucose (HG) dose that induces acute hyperglycemia^21^) a combination of both 2.52 g/kg BHB and 2 g/kg glucose (HG + BHB), or a control solution (saline: 1.116 mg/kg NaCl).

*For bioluminescence imaging, a single* cohort of 12 mice was used for all treatment groups, to adhere to the minimalistic animal sacrifice approach, while allowing for within-mouse comparisons. Each mouse received the same treatment sequence of saline, BHB, HG, and HG+BHB, with each experiment spaced 3–7 days apart. Body weight was measured before each injection to ensure accurate dosing and to monitor the health of the mice (Supplemental Figure S1 A). For consistency, the master mix was prepared on the same day for all mice before each injection.

The timing of the glucose, BHB, or saline injections (4 minutes after CycLuc1 injection) was carefully chosen to allow sufficient time for CycLuc1 to establish its biological effect, thereby ensuring reliable bioluminescence readings.

### Blood glucose and ketone levels

Blood glucose and ketone levels were measured using the GK+ Blood Glucose & Ketone Meter Kit (Keto Mojo). A small blood sample was collected via tail vein puncture from each mouse at two timepoints: immediately before injection (0 min) and 30 minutes post-injection. The blood sample was applied to test strips, and concentrations of glucose and ketones were determined using the meter’s electrochemical sensor. Blood glucose and ketone levels were measured 30 minutes after injections to capture peak systemic concentrations of the administered compounds.

### Photothrombotic stroke model

3-5-month-old C57BL/6 mice were obtained from Jackson Laboratories (#000664) and maintained in standard Association for Assessment and Accreditation of Laboratory Animal Care International (AAALAC)-approved animal care facility with controlled temperature and humidity, a 12-hour light/dark cycle and ad libitum access to water and chow. The photothrombotic stroke method was previously described.^22^ In brief, mice were anesthetized with isoflurane. Hair was removed from the top of the head using a chemical depilatory. The mouse was placed on the surgical platform where the head was cleaned. Mice were given a 5 mg/kg subcutaneous injection of non-steroidal anti-inflammatory drug, Meloxicam, prior to surgery for preemptive analgesia. Mice were injected with a *phosphosensitive dye,* Rose Bengal solution (4 mg/mL injectable saline) by intraperitoneal administration 30 minutes prior to inducing stroke. Rose Bengal is excited upon laser exposure generating reactive oxygen species that damage the endothelial wall, resulting in the formation of a thrombosis and localized vascular occlusion. For the stroke model, a separate cohort of mice was used to assess infarct volume under different treatment conditions. After 15 minutes of Rose Bengal injection, either NaCl control (1.116 mg/kg), BHB (2.52 g/kg), HG (2 g/kg), or both [BHB (2.52 g/kg), HG (2 g/kg)] were injected intraperitoneally 15 minutes before the photothrombotic stroke. The mouse was then placed on a stereotaxic frame to ensure precise targeting of the infarct site, and isoflurane anesthesia (1.5–2%) was maintained throughout the procedure with continuous oxygen supply.

Using aseptic techniques, a 1.5 cm midline incision was made through the skin. The connective tissue covering the skull was removed using small scissors, followed by cleaning the skull surface with a hydrogen peroxide swab. The Bregma was located under the microscope to establish anatomical reference points.

The fiber optic pipette probe was placed 1.7 mm lateral to the midline in the right hemisphere and 10 mm from the surface of the skull. The right hemisphere was illuminated through the intact skull with a 561-nm laser fiber optic illuminator (Coherent Sapphire) with a 3-mm beam diameter for 15 minutes. The laser setting was 45 mW at 38%, and all laser procedures were performed in a class 4 laser safety room with a laser curtain, in compliance with applicable safety regulations. After 15 minutes of illumination, the probe was removed, and the incision was closed with sutures and gently cleaned with chlorhexidine. A thin layer of a first-aid antibiotic with pain relief was applied. Mice were injected with 10 mg/kg of Enrofloxacin to treat systemic bacterial infections. After surgery, mice were kept in a recovery chamber (∼37°C) until euthanasia 24 hours after stroke to assess infarct volume size.

### TTC staining and lesion volume quantification

To assess infarct volume, 2,3,5-Triphenyltetrazolium chloride (TTC, Sigma Aldrich, # T8877) staining was performed as previously described.^22^ Briefly, brains were removed and placed in ice-cold PBS for 5 min on ice. To ensure precise sectioning, brains were positioned in a tissue matrix (Ted Pella, #15050), and sliced into 1 mm thick coronal sections through the brain. Brain slices were then incubated in TTC solution (1% TTC in PBS) for 10 min at 37°C, with slices flipped halfway through incubation to ensure even staining. Once stained, sections were scanned for infarct analysis.

### Scanner-based image acquisition

TTC-stained coronal sections were imaged using an Epson V850 Pro scanner set to professional mode (24-bit color, 1,200 dpi, no color correction, gamma 2.2). To prevent damage or overlap of tissue sections, slices were carefully placed in sequential order (rostral to caudal) between 2 transparencies. The scanner settings included medium exposure and a 1 × 1 pixel densitometer sampling area. A 15 cm ruler was included for pixel-to-distance calibration.

### Image analysis and infarct volume quantification

Image analysis was performed using NIH ImageJ (v1.53K).^23^ To ensure accurate measurements, images were calibrated using a standardized scale by drawing a horizontal line from 1 cm to 2 cm on the 15 cm ruler included in each scan. Under the “Analyze” menu, the “Set Measurements” option was selected, and the distance in pixels was set to 235.0021 pixels corresponding to a known 10 mm length. The pixel aspect ratio was adjusted to 1.0 and the “Global” box was checked to apply setting across all images. For infarct area determination, each coronal section was zoomed to 300% before outlining the infarct using the freshhand tool. The infarct area was measured using “Measure” tool under the “Analyze” menu. Lesion volume per slice was calculated as the average of the front and back side areas multiplied by the slice thickness (1 mm). Total infarct volumes per brain was determined by summing the infarct volumes for all coronal slices of each mouse brain. Detailed method can be found in our previous study.^22^

### Seahorse analysis

Brain tissue samples were obtained as 1 mm biopsies from C57BL/6 mice after euthanasia for *ex vivo* analysis of oxygen consumption rate (OCR) using the Seahorse XF Analyzer (Agilent). Basal OCR measurements were recorded for each group under various conditions, including incubation with normal glucose (NG, 5.5 mM) alone as control, high glucose (HG, 25 mM) alone, and BHB (BHB, 20 mM). Combination treatments of NG and BHB, as well as HG and BHB, were tested. The metabolic modulators were sequentially injected in the following order: Oligomycin (Olig) (1 μM) (Sigma), ATP synthase inhibitor was used to assess ATP-Linked respiration by measuring the decrease in OCR following its addition. FCCP (fluoro-carbonyl cyanide phenylhydrazone, 1 μM), a mitochondrial uncoupler, was added to collapse the proton gradient and measure maximal respiration, representing the full capacity of the electron transport chain. AA/ROT (antimycin A and rotenone, 1 μM each), inhibitors of complex III and complex I, respectively, were used to block mitochondrial respiration completely.

These sequential injections allowed the assessment of key mitochondrial parameters: Basal respiration, ATP production, proton leak, coupling efficiency, maximal respiration, spare respiratory capacity (SRC), non-mitochondrial OCR, and acute OCR response reflecting the immediate mitochondrial response to substrate addition. This protocol is described in Agilent Seahorse XF Cell Mito Stress Test user guide and in our previous study.^24^

### Statistical analysis

Statistical analysis was performed using GraphPad Prism 10. For comparisons between 2 groups with repeated measurements, a paired two-tailed Student’s t-test was used. When comparing more than 2 groups with repeated measures, a repeated measures one-way analysis of variance (ANOVA) followed by Tukey’s post hoc test was applied to adjust for multiple comparisons. For independent group comparisons involving more than 2 conditions, a one-way ANOVA followed by Tukey’s post hoc test was applied. Data are presented as means ± standard deviation (SD). Statistical significance was set at p < 0.05.

## Results

### Acute hyperglycemia decreases ATP flux in the mice brain

**Figure 1.**
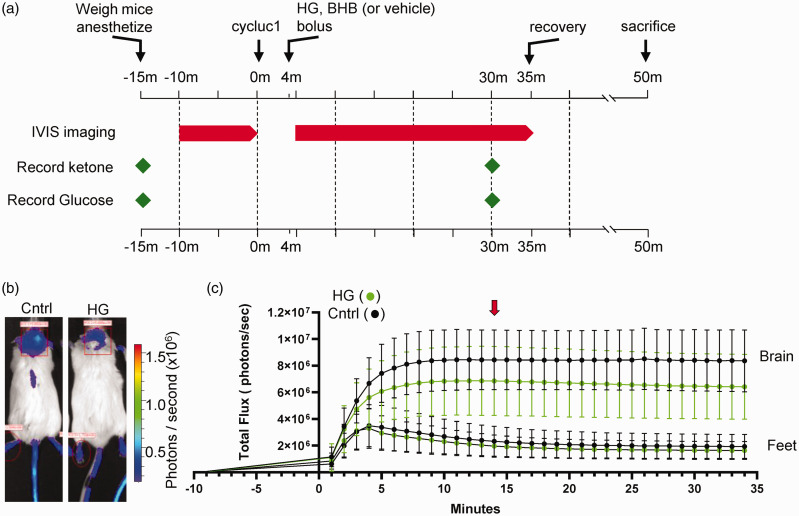
Luciferin-luciferase bioluminescence decreases in response to subcutaneous injections of high glucose (HG). (a) Experimental timeline of luciferin-luciferase bioluminescence imaging experiments. (b) Transgenic mice expressing luciferase enzyme in astrocytes (Gfap promoter), 14 minutes after injection of luciferin substrate and 10 minutes after injection of either NaCl (control) or HG (2 g/kg). Region of interest measured for bioluminescence is shown with red rectangle. Minor expression of luciferase in the skin is observed in tail and feet. The regions of interest over mouse feet, indicated by red ovals, were used as control signals from mice injected with either NaCl or HG and (c) Graphical representation of the bioluminescence emission expressed as flux (photons/sec) *vs*. time in minutes. The red arrow indicates the time of image acquisition at peak flux time. Graphs represent means ± SD.

The adult brain is widely recognized as a glucose metabolizing organ.^25^ To assess the effect of acute hyperglycemia on brain metabolism, we employed bioluminescence imaging experiments to measure ATP-dependent luciferase activity in transgenic Dual Glo mice expressing luciferase in astrocytes (Figure 1(a)). Representative images of mice subsequently injected with either NaCl control (1.16 mg/kg) or HG (2 g/kg) are shown in Figure 1(b). Note the decrease in photon flux in the brain region after HG injections. We found the bioluminescent signals (photons per second) were consistently lower in mice injected with HG compared to control mice (Figure 1(c)). The peak total photon flux occurred approximately 10 minutes post-injection for both HG-injected and vehicle-injected mice, at 6.86 × 10^6^ ± 0.75 × 10^6^ (n = 12) and 8.44 × 10^6^ ± 0.65 × 10^6^ (n = 12), respectively. For comparison with tissue responses outside the brain, we simultaneously monitored photon flux in the foot region. We found no statistical differences in the total flux emitted from feet in mice 10 minutes post-injection with either HG (1.96 × 10^6^ ± 0.23 × 10^6^, n = 12) or saline (2.26 ×10^6^ ± 0.32 × 10^6^, n = 12) (Supplemental Figure S1 B). The data indicates a reduction in brain ATP generation with high glucose.

### β-hydroxybutyrate (BHB) increases ATP flux in the mice brain

**Figure 2.**
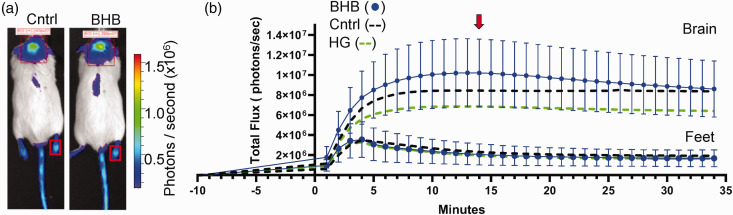
Luciferin-luciferase bioluminescence changes in response to subcutaneous injections of β-hydroxybutyrate (BHB). (a) Transgenic mice expressing luciferase enzyme in astrocytes (Gfap promoter), 14 minutes after injection of luciferin substrate or 10 minutes after injection of either NaCl (control) or BHB (2.5 g/kg). Region of interest measured for bioluminescence is shown with red rectangle. Minor expression of luciferase in the skin is observed in tail and feet. The regions of interest over mouse feet, indicated by smaller red rectangles, were used as control signals from mice injected with either NaCl or BHB and (b) Graphical representation of the bioluminescence emission expressed as flux (photons/sec) *vs*. time in minutes. The red arrow indicates the time of image acquisition at peak flux time. Graphs represent means ± SD.

**Figure 3.**
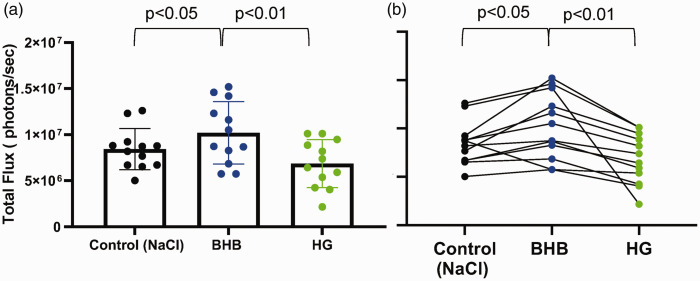
β-hydroxybutyrate (BHB) increases luciferin-luciferase bioluminescence in mouse brain. (a) Bar plot comparing bioluminescence in individual mice 14 minutes after injection of luciferin substrate or 10 minutes post-injection of either NaCl Control, BHB (2.52 g/kg), or HG (2 g/kg). Graph represents means ± SD and (b) Paired data plot showing photon flux measurements for each individual mouse under the three conditions. Each line connects data points corresponding to the same mouse. Each dot represents the photon flux of an individual mouse. Statistical significance between conditions was assessed using repeated measures ANOVA test with Tukey’s multiple comparison test.

We next measured the impact of injecting the ketone BHB on brain metabolism in Dual Glo mice. As above, adult Dual Glo mice were injected with the D-luciferin analog Cycluc1. Four minutes later, mice were injected with BHB (2.52 g/kg), and images were acquired on the Xenogen IVIS system. We observed a higher level of photon flux in the brain region of BHB-injected mice compared to saline-injected mice, which was visually apparent 10 minutes post-injection (Figure 2(a)). Minute-by-minutes changes in total photon flux after BHB injections are presented (Figure 2(b)). For comparison, the total photon flux for each minute has been replotted from Figure 1 for HG (green dashed line) and control mice (black dashed line). The individual photon flux values for each mouse, recorded 10 minutes post-injection of saline, BHB and HG, are presented in Figure 3(a) and (b). Together, these studies suggest that acute BHB treatment may improve ATP flux under both normoglycemic and hyperglycemic conditions.

### *β*-hydroxybutyrate (BHB) sustains brain metabolism in acute hyperglycemic conditions

**Figure 4.**
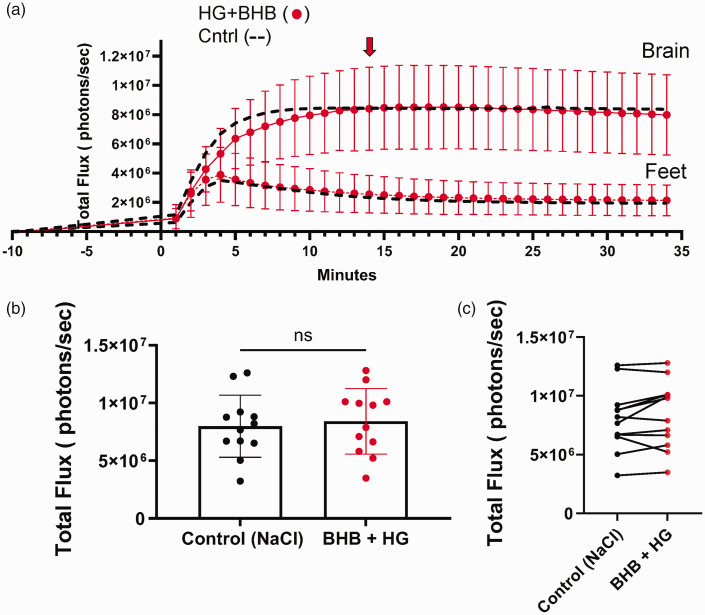
β-hydroxybutyrate (BHB) co-administered with high glucose (HG) maintains bioluminescence at control levels. (a) Bioluminescence of mice expressing luciferase under Gfap promoter (astrocytes). Mice were injected with luciferin substrate, followed, 4 minutes, later by either control (NaCl), HG + BHB. Photon flux was measured every minute post-luciferin injection. BHB (2.52 g/kg) and HG (2 g/kg) were injected together. The red arrow indicates the time of image acquisition at peak flux time. (b) Bar plot comparing photon flux at 14 minutes post-luciferin injection between the control (NaCl) group and the HG + BHB group and (c) Paired data plot showing individual photon flux measurements for each mouse. Each line connects the photon flux values of the same mouse under both conditions, demonstrating within-subject variability. All measurements were acquired on Xenogen IVIS system. Each dot represents an individual mouse, and graphs represent means ± SD. Paired two-tailed t-test indicated no significant difference (ns).

We also measured the impact of simultaneously injecting HG and the ketone BHB on brain metabolism. As above, dual glo mice were injected with CycLuc1 followed 4 minutes later by mixed solution of HG and BHB, the peak photon flux for HG and BHB measured in the brain region (red symbols/line, Figure 4) was comparable to control mice injected with NaCl control alone (black dashed line, Figure 4), albeit with a slower initial rise in photon flux for the first 10 minutes. By minute 14 (equivalent to 10 minutes post-injection of HG+ BHB), photon flux levels were identical to control values. Histogram and paired data plots of brain photon flux for individual mice are presented in Figure 4(b) and (c). No significant differences in photon flux were observed between control and HG + BHB injected mice.

### β-hydroxybutyrate (BHB) ameliorates blood glucose level amid acute hyperglycemia setting

**Figure 5.**
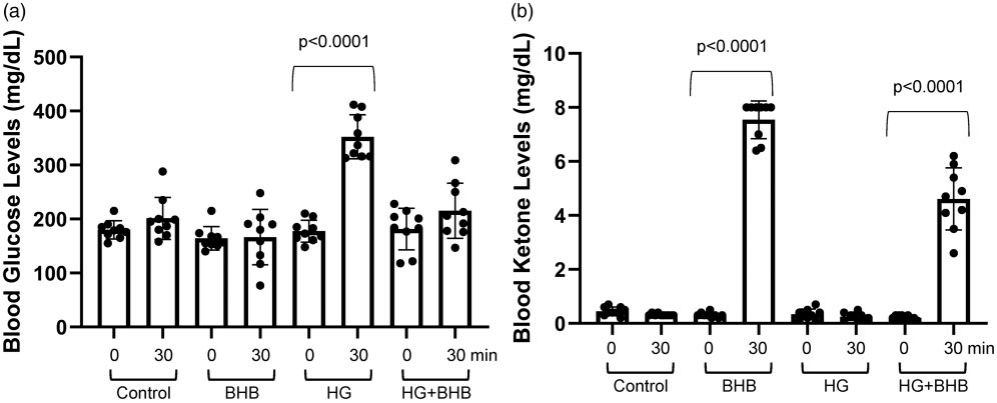
Blood glucose and ketone concentrations after subcutaneous injection of high glucose (HG) and β-hydroxybutyrate (BHB). (a) Blood glucose concentrations in mice before (0 min) and after (30 min) injections of NaCl control, BHB (2.52 g/kg), HG (2 g/kg) and [BHB (2.52 g/kg) +HG (2 g/kg)] together as indicated and (b) Blood ketone concentrations in mice before (0 min) and after (30 min) injections of NaCl control, BHB (2.52 g/kg), HG (2 g/kg) and [BHB (2.52 g/kg) +HG (2 g/kg)] as indicated. Each symbol is an individual mouse. Data are presented as means ± SD. Statistical significance was determined using Tukey’s multiple comparison test.

We measured blood levels of HG and BHB in mice, both before and 30 minutes after subcutaneous injections of saline, HG, BHB or HG+BHB. As expected, blood glucose levels were not affected by saline or BHB injections (Figure 5(a)). However, the average blood glucose significantly increased (p < 0.0001) from 177.6 ± 6.8 mg/dl (n = 9) at 0 minutes to 352.3 ± 13.6 (n = 9), 30 minutes after mice had been injected with HG (Figure 5(a)). Blood glucose levels were not significantly changed when HG and BHB were injected together. Saline and HG injections had no effect on blood ketone levels, while BHB injections significantly increased (p < 0.0001) the blood ketone levels to 7.54 ± 0.23 mM (n = 9) 30 minutes post-injection (Figure 5(b)). When BHB was co-injected with HG, ketone levels rose to 4.61 ± 0.38 mM (n = 9).

### Effects of β-hydroxybutyrate (BHB) on mitochondrial respiration parameters under normoglycemic and hyperglycemic conditions

**Figure 6.**
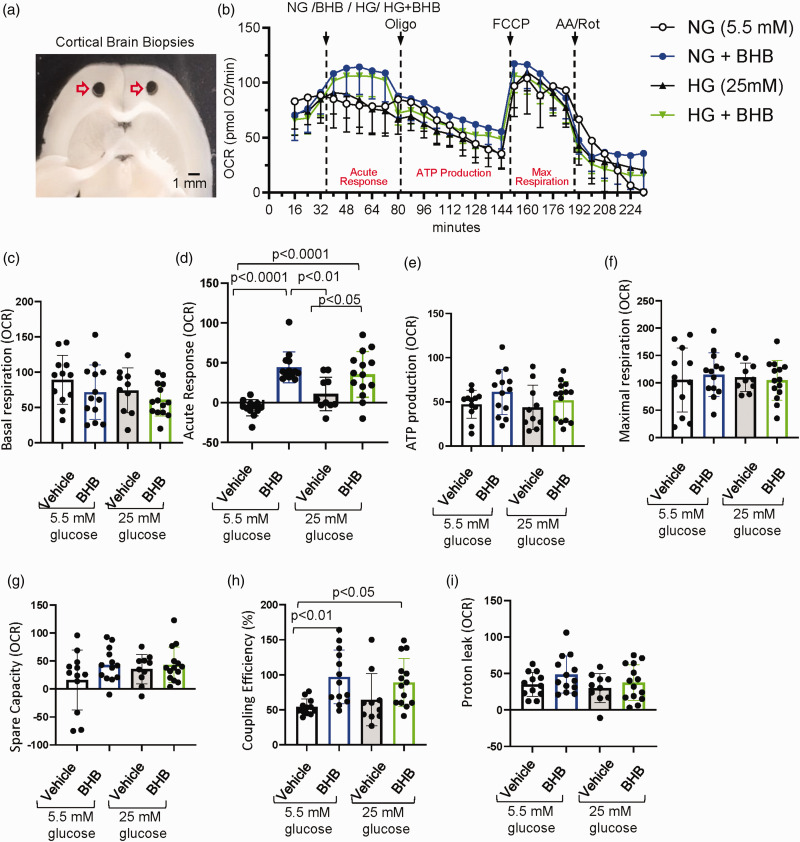
Cortical brain biopsy respiration analysis under normoglycemic and hyperglycemic conditions with and without β-hydroxybutyrate (BHB). (a) Microscopic images of cortical mouse brain biopsies retrieved after sacrifice and used for *ex vivo* Seahorse analysis. (b) Oxygen consumption rate (OCR), measured in pmol O_2_/min, plot showing dynamic changes in oxygen consumption across treatments, including normal glucose (NG, 5.5 mM glucose), NG + 20 mM BHB, high glucose (HG, 25 mM glucose), and HG + BHB. OCR values were recorded for 232 minutes, capturing baseline respiration, sequential injection responses, and acute OCR changes following substrate administration. (c) Basal respiration, calculated as the last OCR measurement before the acute injection (measurement # 3) minus non-mitochondrial respiration (minimum rate after AA/Rot injection). (d) Acute response, calculated as the difference between the fourth OCR measurement after substrate injection (measurement #7) and the last basal OCR measurement before acute injection (measurement #3). (e) ATP production, calculated as the difference between last OCR measurement before oligo injection (measurement # 9) and minimum rate measurement after oligo injection (measurement # 18). (f) Maximal respiration, calculated as the maximal OCR after FCCP injection (measurement # 20) minus non-mitochondrial respiration. (g) Spare respiratory capacity (SRC), calculated as maximal respiration minus basal respiration (h) Coupling efficiency, calculated as ATP production/Basal respiration ×100 and (i) Proton leak, calculated as the minimum OCR after oligo injection (measurement # 18) minus non-mitochondrial respiration. Data was acquired on a Seahorse XF analyzer (Agilent). 24 well system from 4 mice, replicates counts are as such: 12 NG, 10 HG, 13 NG+BHB and 14 HG+BHB. Data are presented as means ± SD. Statistical significance was determined using One-way ANOVA test followed by Tukey’s multiple comparison test. AA/Rot: antimycin A and rotenone; FCCP: carbonyl cyanide-4 (trifluoromethoxy) phenylhydrazone; Oligomycin (Oligo).

Oxygen consumption rate (OCR) measurements of *ex vivo* brain tissue (1 mm biopsies), which indicate mitochondrial activity, are shown in Figure 6(a). OCR dynamics over time for all treatments (NG, NG+BHB, HG, HG+BHB) are illustrate in Figure 6(b), highlighting the mitochondrial response to the sequential addition of modulators and substrates.

Basal respiration, representing the OCR in the absence of additional substrates or stressors, showed no significant differences between groups. The normal glucose (NG) control group exhibited a basal OCR of 89.25 pmol O_2_/min, compared to 71.77 pmol O_2_/min in the NG+BHB group, 74.3 pmol O2/min in the HG group and 61.14 pmol O_2_/min in the HG+BHB group (Figure 6(c)). These findings suggest that BHB does not alter basal mitochondrial activity.

Acute OCR response, indicating the immediate reaction of mitochondria to the addition of modulators, showed significant increases in NG+BHB (44.46 pmol O_2_/min) and HG+BHB (35.57 pmol O_2_/min) groups compared to NG (-6.5 pmol O_2_/min) and HG (10.9 pmol O_2_/min) controls (p < 0.05) (Figure 6(d)).

ATP production, indicating mitochondrial efficiency in converting substrates into usable energy, showed a trend toward an increase with BHB in NG (61.15 pmol O_2_/min) compared to NG controls (47.17 pmol O_2_/min), though the difference was not statistically significant. In HG conditions, ATP production remained consistent between HG (43.7 pmol O_2_/min) and HG+BHB (51.79 pmol O_2_/min) groups (Figure 6(e)).

Maximal respiration, which measures the full capacity of the electron transport chain under uncoupled conditions, showed no significant differences between groups (Figure 6(f)). Spare respiratory capacity (SRC), indicating the difference between maximal respiration and basal respiration and reflecting the ability of mitochondria to respond to increased energy demands, also showed no significant differences across groups (Figure 6(g)). However, the coupling efficiency, indicating the proportion of oxygen consumption used for ATP production relative to total oxygen consumption, was significantly improved with BHB treatment. Under NG conditions, coupling efficiency increased from 54.1% in controls to 96.95% with BHB, and under HG conditions, coupling efficiency increased from 64.47% to 89.11% (Figure 6(h)). Proton leak, representing uncoupled respiration, increased (but not significantly) with BHB treatment in both NG and HG conditions (Figure 6(i)). These findings indicate that BHB enhances mitochondrial efficiency and acute responsiveness without altering basal or maximal respiration.

### Acute β-hydroxybutyrate (BHB) reduced infarct volume in a thrombotic stroke model

**Figure 7.**
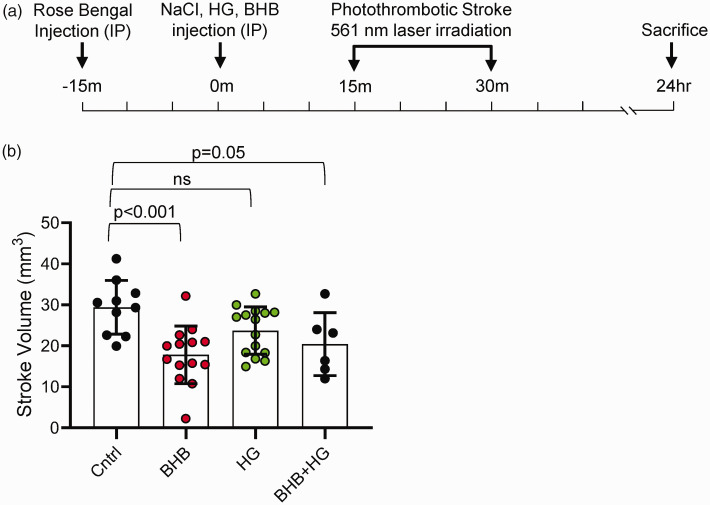
Effect of β-hydroxybutyrate (BHB) on brain stroke infarct volume. (a) Schematic of the experimental design. Mice were injected with Rose Bengal solution 30 minutes before stroke induction. Treatments [NaCl (n = 10), BHB (n = 14), HG (n = 15), or HG + BHB (n = 6)] were administered intraperitoneally 15 minutes before stroke induction. The photothrombotic stroke was induced using a 561 nm laser for 15 minutes, and mice were sacrificed 24 hours post-stroke and (b) Extracted brains were immediately sectioned and stained with TTC to measure Infarct volumes. Data are presented as means ± SD. Statistical significance was determined using one-way ANOVA test followed by Tukey’s multiple comparison test.

In order to establish a functional link between metabolism and brain health, we investigated the acute effects of acute BHB in a photothrombotic stroke model, measuring infarct volume as a functional outcome (Figure 7(a)). Mice treated with BHB demonstrated a significant reduction in infarct volume compared to controls. The average infarct volume was 29 ± 2.1 mm³ in the control (NaCl) group, which was reduced to 18 ± 1.9 mm³ in the BHB group (p < 0.001). Mice treated with HG alone had an infarct volume of 24 ± 1.5 mm³, while the combination of HG and BHB resulted in a smaller infarct volume of 20 ± 3.1 mm³ (p = 0.05 compared to NaCl control group) (Figure 7(b)). These results provide evidence of a functional acute benefit of BHB, demonstrating its cerebroprotective effects both under normal and hyperglycemic conditions.

## Discussion

The major goal of our multi-disciplinary study was to identify a mechanism by which a ketogenic diet may be beneficial to the brain. Our hypothesis was that ketone bodies can mitigate acute metabolic stress on the brain. The data in mice presented above indicate that acute hyperglycemia induces a decrease in ATP levels within the brain, while BHB supplementation exhibits promising effects on brain metabolism under such conditions. Specifically, we found that BHB increases ATP flux in the brain and ameliorates both brain metabolism and blood glucose levels during acute hyperglycemia.

BHB may also enhance glucose utilization and insulin sensitivity in non-diabetic individuals, as evidenced by studies such as Myette-Côté et al.,^26^ which demonstrated an improved insulin sensitivity index and reduced glucose area under the curve following BHB supplementation. This highlights the possible ability of BHB to complement insulin action by promoting glucose regulation and metabolic balance.

We also investigated the impact of BHB on mitochondrial function using OCR analysis. We found that BHB significantly enhanced coupling efficiency in both normoglycemic and hyperglycemic conditions, indicating improved mitochondrial efficiency. This increase in coupling efficiency was not accompanied by a significant change in ATP production, suggesting that BHB improves mitochondrial performance by minimizing inefficiencies (e.g. non-productive respiration) rather than directly increasing energy output. This is also consistent with the idea that cellular ATP production is demand-driven and may not rise unless energy requirements increase.^27,28^ An important caveat of this interpretation is that these measurements were performed on brain biopsies, where the heterogeneous metabolic activity of neurons, astrocytes, and other brain cells,^29^ along with the high basal metabolic rate of brain tissue,^30^ could mask detectable changes in cell-specific ATP production.

Additionally, BHB increased the acute OCR response following substrate addition, particularly under HG conditions, suggesting a rapid and adaptive mitochondrial response. This enhanced acute OCR response may indicate that BHB primes mitochondria for metabolic flexibility, enabling a quicker adjustment to substrate availability and metabolic stress. These findings align with prior studies, such as Shimazu et al.,^31^ which demonstrated that BHB enhances energy efficiency and reduces oxidative stress in neuronal models.

The mild and potential increase in proton leak, observed with BHB treatment, may reflect a regulatory mechanism to mitigate reactive oxygen species (ROS) production, possibly by dissipating excess mitochondrial membrane potential, as suggested in other studies.^32–34^ These adaptations suggest an acute role of BHB in improving efficient energy utilization while enhancing mitochondrial resilience even under metabolic stress.

In addition, our photothrombotic stroke data reveal that BHB significantly reduced infarct size, providing direct functional evidence of its cerebroprotective effects.

### Acute hyperglycemia alters brain metabolism even in the absence of diabetes

Energy metabolism in the brain is a complex process that involves the regulation of glucose uptake and utilization, as well as the metabolism of phospholipids. Glucose is a crucial energy substrate for the brain, and its uptake is tightly regulated to meet the high energy demands of the brain.^29,35^ The brain’s reliance on glucose as a primary energy source is well-established, and alterations in glucose metabolism can have significant effects on brain function.^36^

Here we highlight that acute hyperglycemia could alter the brain’s metabolic dynamics even in the absence of diabetes. A study in humans employing non-invasive magnetic resonance techniques showed that ingesting 50 g glucose decreased global cerebral metabolic rate of oxygen in a time-dependent pattern, which was accompanied by a reduction in oxygen extraction fraction and metabolism of oxygen.^37^

Our Seahorse data demonstrated that BHB enhanced mitochondrial oxygen consumption even under hyperglycemic potential stress, as evidenced by improved coupling efficiency and acute OCR response. These mitochondrial adaptations suggest that BHB provides an acute metabolic advantage in balancing energy demands and limiting hyperglycemia-induced inefficiencies.

### BHB is a brain-friendly energy substitute that enhances oxygen consumption rate

BHB has gained attention for its potential as an alternative energy source in the absence of glucose. Studies have shown that BHB not only serves as an energy source but also exhibits neuroprotective properties in models of neurodegenerative diseases like Parkinson’s, Huntington’s, and Alzheimer’s.^38–41^

Our study is the first to investigate the exogenous BHB effect under normoglycemia and acute hyperglycemia and provides an explanation of how BHB could protect the brain by improving mitochondrial efficiency.

Our data indicate that ATP flux under BHB-treatment is increased and returns to control levels over time, suggesting a transient metabolic boost provided by BHB under acute conditions. Conversely, HG-treatment exhibited a consistent decrease in flux, indicating a sustained negative impact on mitochondrial function. The HG + BHB treatment maintained a control flux level, but this was associated with a slight decrease in ATP flux over time, suggesting the beneficial effects of BHB may be temporary. This observation emphasizes the need for studies exploring prolonged BHB exposure or repeated dosing to determine its potential for sustained metabolic benefits.

BHB’s anti-inflammatory and antioxidant properties may help mitigate oxidative stress and inflammation associated with diabetes, thereby enhancing overall metabolic health and lowering the risk of diabetes-related complications.^41–43^ We observed that BHB increases brain OCR in normal or high glucose conditions; this may be partly explained by the effect of BHB on astrocytic glucose consumption, as shown in Valdebenito et al. (2015),^44^ where BHB inhibits glucose uptake in astrocytes. Modulation of astrocytic glucose metabolism may limit the immediate impact of high glucose levels in the brain, shifting energy metabolism toward BHB utilization and protecting against hyperglycemia-induced metabolic alterations. Prior studies have demonstrated that 30 mg/kg of BHB administered to mice post brain-ischemia-reperfusion improved mitochondrial function and enhanced OCR.^45^ Interestingly, BHB was found to inhibit apoptosis in astrocytes exposed to high glucose concentration.^46^

In addition to its metabolic effects, our study demonstrated that BHB significantly reduced infarct size in a photothrombotic stroke model. This model is particularly relevant as it induces localized ischemic injury and replicates the acute metabolic and mitochondrial stress that occurs during stroke,^22,47^ providing a platform to evaluate the immediate effects of metabolic modulators such as BHB.

Our data are supported by recent studies showing that BHB administration via drinking water for 14 days before stroke improved cognitive and motor recovery post-stroke through activation of the nuclear factor erythroid 2-related factor 2 (Nrf2)/antioxidant response element (ARE) pathway.^48^ Furthermore, post-stroke BHB delivery has been associated with reduced peri-infarct glucose uptake, ROS production and astrogliosis, alongside improved neuronal functioning.^49^ Research has also shown that acute BHB administration immediately after reperfusion in a mouse model of transient ischemia enhanced mitochondrial respiration, particularly through improved complex I and II activity.^45^ Interestingly, our results demonstrate that the neuroprotective effect of BHB, even when administered acutely before ischemia, underscores its translational potential in mitigating brain injury during acute metabolic and ischemic stress.

### Exogenous BHB, at moderate concentrations, has positive physiological effects

We and others demonstrate that exogenous BHB treatment regulates blood sugar levels by providing an alternative fuel source for insulin-resistant cells, potentially improving insulin sensitivity and reducing insulin resistance.^50–52^ Exogenous ketosis achieved without carbohydrate restriction has been shown to reduce blood glucose levels effectively. For instance, infusion of BHB has been observed to lower blood glucose levels in both animals^52–55^ and human subjects.^56^ However, it is noteworthy that BHB levels increase in correlation with the severity of diabetic ketoacidosis.^57^ The consideration of BHB’s safe-dose administration is critical to procure brain and systemic safety.

Hence, it is important to appreciate that a low concentration of exogenous BHB administered to mice has a significant protective effect on the brain under both normal and acute hyperglycemic conditions. This safe, low-level BHB administration is reiterated with other studies demonstrating the potential reversal of type 2 diabetes through very low carbohydrate or ketogenic diets.^52^ Thus, monitoring ketone levels during a ketogenic diet is crucial to ensure the diet’s effectiveness and safety.

Limitations of this study include a lack of direct measures of ATP and that only astrocytes were assessed for the impact of hyperglycemia. In future studies we plan to perform spatial metabolomics to discern ATP, adenosine diphosphate (ADP) and adenosine monophosphate (AMP) *in situ* and co-localize with specific cell types.

Additionally, our findings reflect acute events, which may differ under chronic conditions. Focusing on acute metabolic and neuroprotective effects, including in the stroke model, limited the possibility of conducting comprehensive functional assessments in addition to infarct volume. Moreover, chronic metabolic dysregulation, such as that seen in diabetes, involves long-term adaptations in glucose transporters and mitochondrial function, which could impact BHB’s metabolism and efficacy. Future studies extending the duration of experiments while integrating longitudinal behavioral or neurological evaluations or employing models of chronic hyperglycemia are necessary to explore the translational relevance of our findings to diseased states.

In conclusion, our study provides a firm foundation for future studies that investigate BHB as a variable in determining brain metabolism protection especially in the setting of a ketogenic diet that is mixed with low or high carbohydrate content.

## Supplemental Material

sj-pdf-1-jcb-10.1177_0271678X251334222 - Supplemental material for **β**-hydroxybutyrate enhances brain metabolism in normoglycemia and hyperglycemia, providing cerebroprotection in a mouse stroke model Supplemental material, sj-pdf-1-jcb-10.1177_0271678X251334222 for **β**-hydroxybutyrate enhances brain metabolism in normoglycemia and hyperglycemia, providing cerebroprotection in a mouse stroke model by Deborah M Holstein, Afaf Saliba, Damian Lozano, Jiwan Kim, Kumar Sharma and James D Lechleiter in Journal of Cerebral Blood Flow & Metabolism

## Data Availability

All data generated or analyzed during this study are included in the figures and supplementary materials of this manuscript. Additional clarification or access to raw data are available upon reasonable request from the corresponding author.
