# Supplementary material for: β-hydroxybutyrate enhances brain metabolism in normoglycemia and hyperglycemia, providing cerebroprotection in a mouse stroke model
Source: J Cereb Blood Flow Metab. 2025 Apr 12;45(8):1493–506. doi: 10.1177/0271678X251334222 (PMC11993551; doi:10.1177/0271678X251334222)
Supplement: sj-pdf-1-jcb-10.1177_0271678X251334222 - Supplemental material for β-hydroxybutyrate enhances brain metabolism in normoglycemia and hyperglycemia, providing cerebroprotection in a mouse stroke model [file sj-pdf-1-jcb-10.1177_0271678X251334222.pdf]

Holstein D M and Saliba A et al.

Supplemental File

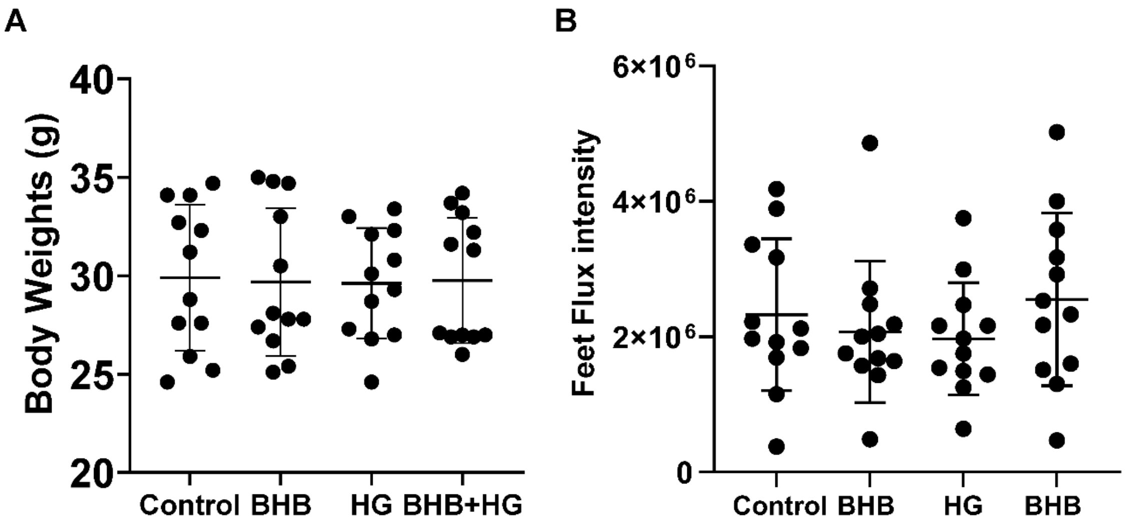

Supplemental Figure S1. Luciferin-luciferase bioluminescence imaging experiments mice body weights and feet flux intensity. (**A**) Mice body weights recorded before bioluminescence imaging. (**B**) Total flux emitted from feet in mice 10 minutes post-substrate injection (peak). Data are presented as mean ± SD. Statistical significance was determined using repeated measures one-way ANOVA test followed by Tukey's multiple comparison test showing no significant changes.
